# What is the advantage of rectal amputation with an initial perineal approach for primary anorectal carcinoma?

**DOI:** 10.1186/s12893-020-0683-5

**Published:** 2020-02-03

**Authors:** Kimihiko Funahashi, Mayu Goto, Tomoaki Kaneko, Mitsunori Ushigome, Satoru Kagami, Takamaru Koda, Yasuo Nagashima, Kimihiko Yoshida, Yasuyuki Miura

**Affiliations:** Department of General and Gastroenterological Surgery, Toho University Omori Medical Center, 6-11-1 Omorinishi Otaku, Tokyo, 143-8541 Japan

**Keywords:** Rectal amputation with an initial perineal approach, Sphincter-preserving resection, Low-lying rectal cancer near the anus

## Abstract

**Background:**

Rectal amputation (RA) remains an important surgical procedure for salvage despite advances in sphincter-preserving resection, including intersphincteric resection. The aim of this study was to compare short- and long-term outcomes of RA with an initial perineal approach to those of RA with an initial abdominal approach (conventional abdominoperineal resection (APR)) for primary anorectal cancer.

**Methods:**

We retrospectively analyzed the short- and long-term outcomes of 48 patients who underwent RA with an initial perineal approach (perineal group) and 21 patients who underwent RA with an initial abdominal approach (conventional group).

**Results:**

For the perineal group, the operation time was shorter than that for the conventional group (313 vs. 388 min, *p* = 0.027). The postoperative complication rate was similar between the two groups (43.8 vs. 47.6%, *p* = 0.766). Perineal wound complications (PWCs) were significantly fewer in the perineal group than in the conventional group (22.9 vs. 57.1%, *p* = 0.006). All 69 patients underwent complete TME, but positive CRM was significantly higher in the conventional group than in the perineal group (0 vs. 19.0%, *p* = 0.011). There were no significant differences in the recurrence (43.8 vs. 47.6%, *p* = 0.689), 5-year disease-free survival (63.7% vs. 56.7%, *p* = 0.665) and 5-year overall survival rates (82.5% vs. 66.2%, *p* = 0.323) between the two groups.

**Conclusion:**

These data suggest that RA with an initial perineal approach for selective primary anorectal carcinoma is advantageous in minimizing PWCs and positive CRMs. Further investigations on the advantages of this approach are necessary.

## Background

In rectal cancer surgery, the minimization of local recurrence and preservation of sphincter function are a concern. Historically, lower rectal cancer near the anus was not treated with anything other than rectal amputation (RA). Intersphincteric resection (ISR) has been reported as a sphincter-preserving resection procedure for lower rectal cancer, and ISR makes preservation of the anus possible for patients with low-lying rectal cancer near the anus. However, we often require RA as a salvage treatment when tumor invasion of the external anal sphincter muscle is suspected during a sphincter-preserving resection by super-low anterior resection and ISR. RA remains an important surgical procedure for rectal cancer despite advances in sphincter-preserving resection.

In RA in which the distal rectum and anal sphincter complex are completely removed, we usually start with an abdominal approach to ligate the inferior mesenteric vessels and mobilize the left-side colon. We dissect the rectum as far as possible toward the pelvic floor to facilitate the perineal dissection. Additionally, when combined resection of the sacrum is required for advanced local rectal cancer or when perineal dissection is performed for a large tumor, repositioning, such as to the prone jackknife position, is considered occasionally. Subsequently, we approach the perineum to remove the anorectum, including the distal rectum.

Recently, the number of laparoscopic surgeries for not only colon cancer but also rectal cancer has increased annually, although two randomized controlled trials, the ALaCaRT [1] and the ACOSOG Z6051 trials [2], failed to show the superiority of laparoscopic surgery compared to open surgery for oncologic outcomes. More recently, the use of an alternative surgical technique with an initial transanal approach for patients with a narrow pelvis and/or prostatic hypertrophy to achieve complete total mesorectal excision (TME) has increased [3–6]. We occasionally need to convert from sphincter-preserving resection to RA because of unexpected invasion of the external anal sphincter muscle. Perineal dissection of the anorectum prior to the transabdominal maneuver must be reasonable in a series of procedures. Additionally, this approach might facilitate the resection of tumors with complete TME and negative circumferential resection margin (CRM) for patients with a narrow pelvis and bulky tumors, even in laparoscopic surgery. However, there has been no investigation on the feasibility of RA with an initial perineal approach and a retrograde anorectum dissection for primary anorectal cancer.

The aim of this study was to compare the short- and long-term outcomes of RA using two different approaches, an initial perineal approach and an initial conventional abdominal approach, in primary anorectal carcinoma patients.

## Methods

### Patients

**Fig. 1:**
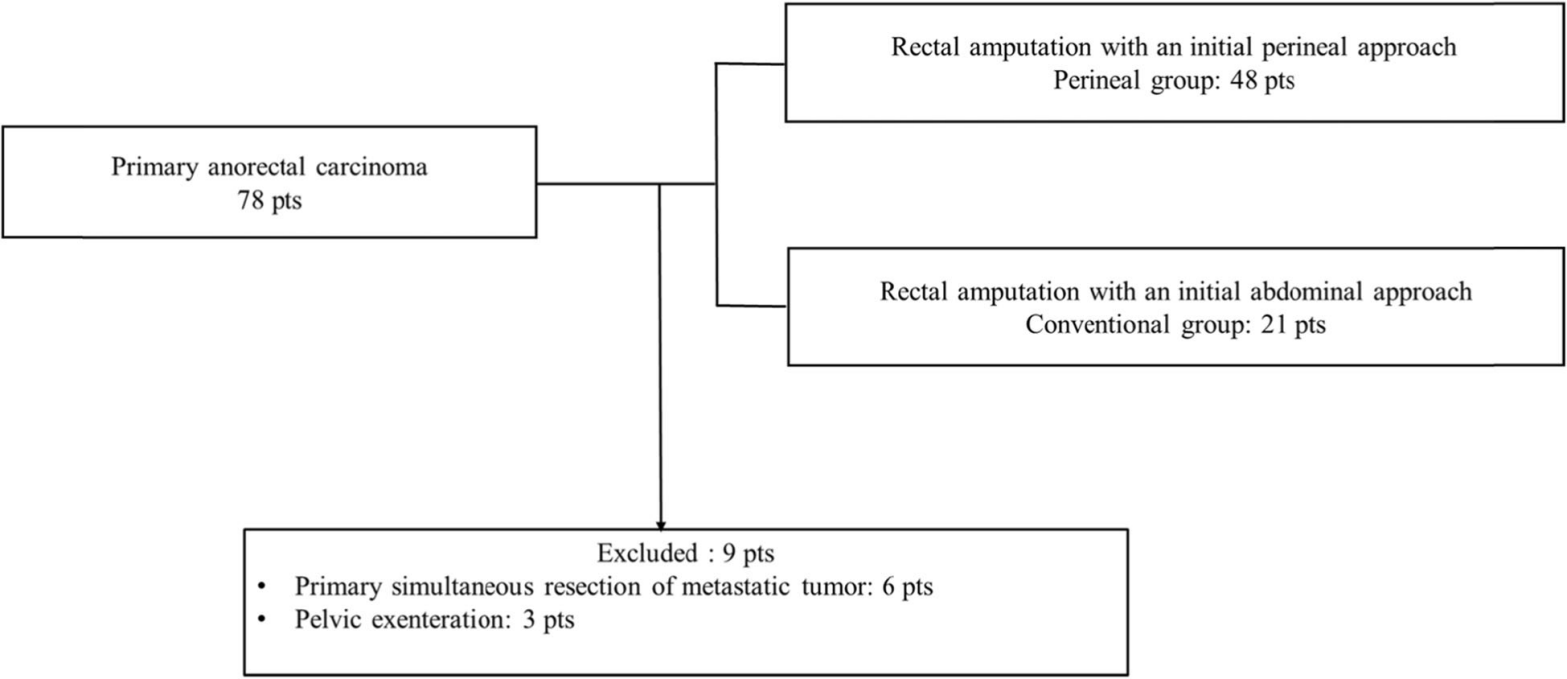
Outcome in 78 patients who underwent RA for patients with primary anorectal carcinoma according to the different surgical approaches

Between January 2004 and December 2014, 78 patients underwent RA for primary anorectal carcinoma in Toho University Omori Medical Center. We excluded nine patients who required simultaneous resection of metastatic tumors in the liver and/or lung and pelvic exenteration for primary tumors invading the prostate or vagina/uterus. Finally, we divided the 69 patients with RA into two groups according to the different surgical approaches; 48 patients were assigned to an RA with an initial perineal approach group (perineal group) and 21 patients were assigned to an RA with an initial abdominal approach group (conventional group) (Fig. 1). The short- and long-term outcomes were compared between the two groups. The surgical RA approach was chosen according to the surgeon’s preference.

This study was approved by the Toho University Omori Medical Center Ethics Committee (M19031). Informed consent was obtained from all individual participants included in the study.

### Technique of rectal amputation with an initial perineal approach

RA was performed with the patient in the Lloyd-Davies position by a single team.

For the perineal group, we first create an elliptical incision from the midpoint of the perineal body in males or the posterior vaginal introitus in females to a point midway between the coccyx and the anus. The incision was continued through the subcutaneous tissue into the ischiorectal fat using electrocautery. Second, we separated the anococcygeal raphe at the posterior aspect and then cut along the avascular holy plane toward the recto-sacral ligament originating from the presacral parietal fascia at the S2 to S4 level. Third, at the anterolateral aspect, we removed the branch of the levator ani muscle that attaches to the anus.

At the anterior aspect, we detached both the transverse perineal muscle and the rectourethralis muscle and then dissected along the posterior wall of the prostate or the vagina to the peritoneal refraction. The goal of perineal dissection was to dissect to the recto-sacral ligament at the posterior aspect and the peritoneal reflection at the anterior aspect transperineally.

Finally, the perineal wound was irrigated well using saline and closed using absorbable sutures. The subcutaneous fat was subsequently reapproximated at the midline using absorbable sutures, and the skin was reapproximated using interrupted monofilament sutures in a vertical mattress fashion.

**Fig. 2:**
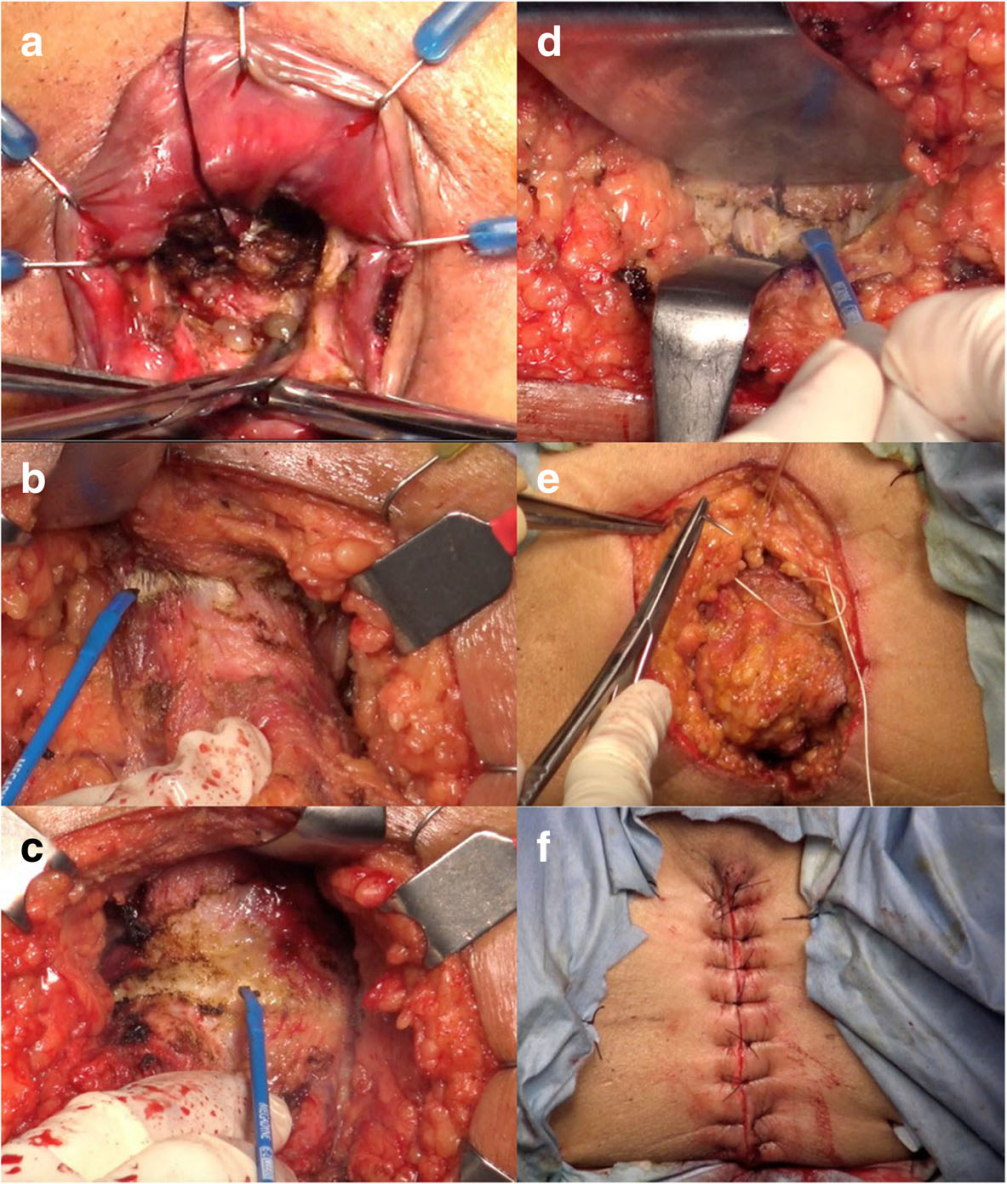
Technique of rectal amputation with an initial perineal approach. In this patient, conversion from intersphincteric resection to rectal amputation was required because mucinous adenocarcinoma invasion was suspected on the dissected plane between the internal and external anal sphincter muscle (**a**). At the anterior aspect, we detached both the transverse perineal muscle and the rectourethralis muscle and then dissected along the posterior wall of the prostate to the peritoneal refraction (**b** and **c**). At the posterior aspect, we separated the anococcygeal raphe and then cut along the avascular holy plane toward the rectosacral ligament originating from the presacral parietal fascia (**d**). Finally, the perineal wound was sufficiently irrigated with saline and closed with absorbable sutures. The subcutaneous fat was subsequently reapproximated at the midline with absorbable sutures, and the skin was reapproximated with interrupted monofilament sutures in a vertical mattress fashion (**e** and **f**)

For the abdominal step in laparoscopic surgery, we usually used five ports: a 12-mm umbilical port for the laparoscope, a 12-mm port and a 5-mm port in the right abdomen for the operator and two 5-mm ports for the assistant to perform retraction. First, we ligated the inferior mesenteric vessels at a high level and removed the left-side colon laparoscopically. Preserving the hypogastric nerves, we dissected the rectum toward the pelvic floor to connect to the plane dissected in the perineal maneuver and then removed the anorectum with the tumor from the pelvic floor. Second, we extended the wound at the umbilicus and then removed the specimen from that location. After resection of the tumor and the regional lymph nodes, we created a colostomy through the extraperitoneum at the preoperatively marked stoma site. Finally, a 19-French round drain was usually laparoscopically placed at the pelvic floor via the left lower quadrant port site (Fig. 2).

### Perineal wound complications

In this study, perineal wound complication (PWC) was defined as the following: wound dehiscence; local infection defined as erythema; purulent discharge; delayed wound healing; and abscess, fistula or ulcer formation within 30 days after surgery. PWC was diagnosed by two surgeons.

### Postoperative follow-up

We followed patients after surgery as follows: blood tests including carcinoembryonic antigen (CEA) and carbohydrate antigen (CA 19–9) were performed every 3 months. Also, computed tomography (CT) or/and abdominal ultrasonography were performed every 3 months in the first 3 years and every 6 months thereafter to evaluate cancer recurrence. In this study, local recurrence was defined as any recurrence that was diagnosed or suspected in the pelvis, either alone or other metastases.

### Statistical analysis

Quantitative data are reported as the median (range). The Mann-Whitney U test was used to compare the continuous variables, and chi-square or Fisher’s exact tests were used to compare the discrete variables. All data were entered in a computer database and analyzed using the Statistical Package for the Social Sciences (SPSS) for Windows software program, version 9.02 (SAS Institute Inc., Cary, NC, USA). Differences were considered significant for *p* values < 0.05.

## Results

### Patient characteristics

The patient characteristics are shown in Table 1. There were 33 males and 15 females in the perineal group and 18 males and 3 females in the conventional group. All of the patients with anorectal cancer underwent pelvic magnetic resonance imaging (MRI) and/or pelvic CT for preoperative assessment. Preoperative chemoradiation therapy (pre-CRT) with TS-1 and neoadjuvant chemotherapy was administered to 7 patients with advanced anorectal cancer. According to the pelvic CT and pelvic MRI images, we chose RA for 20 patients with clinical stage T4 cancer. Although we planned to perform only sphincter-preserving resection for 40 patients with clinical stage T3 cancer using intersphincteric resection, we performed RA because tumor invasion of the external anal sphincter muscle and/or the levator ani muscle was suspected during the sphincter-preserving resection. The surgical approach (initial perineal or initial abdominal) for RA was chosen according to the surgeon’s preference. RA was performed laparoscopically for 12 patients in the perineal group and for 4 patients in the conventional group, respectively.

**Table 1:**
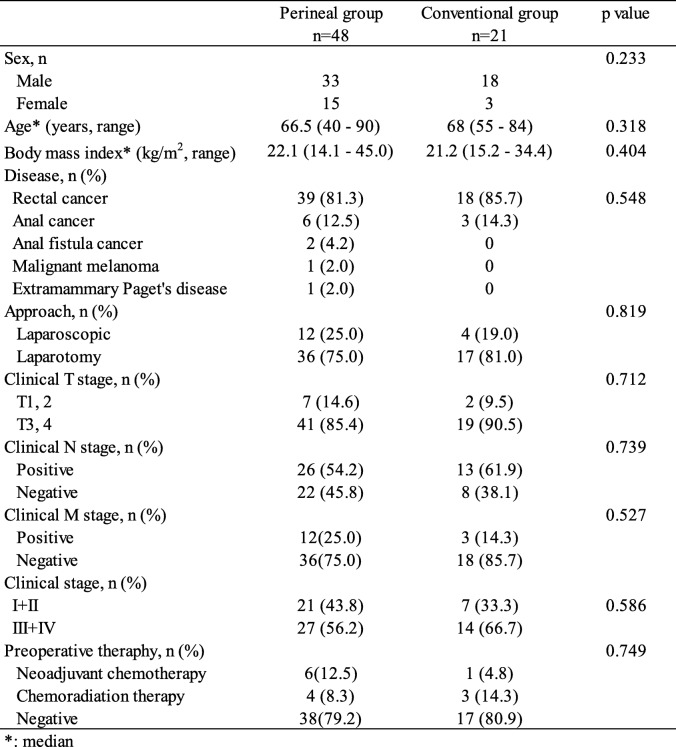
Patient characteristics

There was no difference in sex, age, body mass index, surgical approach (laparotomy vs. laparoscopic surgery), clinical T, N and M stage or preoperative therapy between the two groups.

### Surgical outcomes

Surgical outcomes are shown in Table 2. The median operation time for the perineal group and conventional group was 313 min (range, 144–719 min) and 388 min (range, 212–663 min), respectively. A significant difference was observed between the two groups (*p* = 0.027). There was no difference in the median bleeding volume, at 520 ml (range, 27–2598 ml) in the perineal group vs. 454 ml (230–6134 ml) in the conventional group (*p* = 0.234), between the two groups.

**Table 2:**
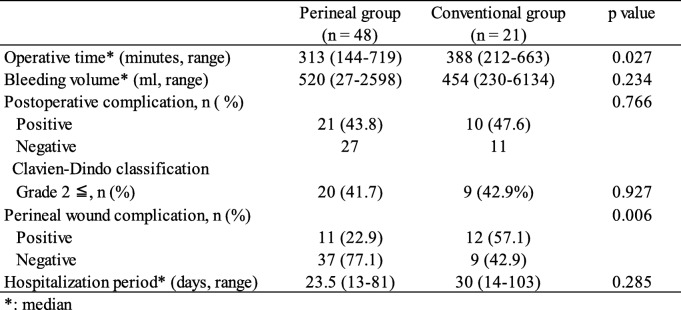
Surgical outcomes

Postoperative surgical complications occurred in 31 (44.9%) of the 69 patients in this study. The postoperative surgical complication rates of the perineal and conventional groups were 43.8% (21 patients) and 47.6% (10 patients), respectively. There was no difference between the two groups (*p* = 0.766). Additionally, postoperative surgical complications rated as grade 2 and higher than grade 2 according to the Clavien-Dindo classification occurred in 20 (41.7%) patients in the perineal group and 9 (42.9%) patients in the conventional group. Neurogenic bladder occurred frequently in both groups (22.9% vs. 28.6%, Table 3), and there was no significant difference between the two groups (*p* = 0.927). The median hospitalization period for the perineal group was 23.5 days (range, 13–81 days), which was shorter than the 30.0 days (range, 14–103 days) for the conventional group; however, the difference was not significant (*p* = 0.285).

**Table 3:**
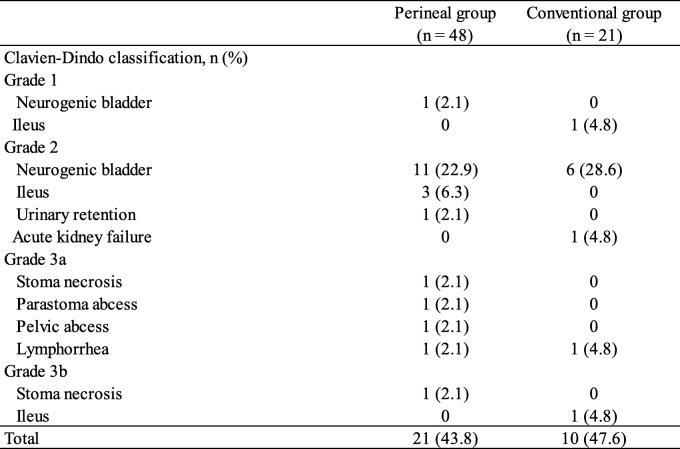
Postoperative complications

Of the 69 patients, 23 (33.3%) patients developed a PWC. The PWC rate in the perineal group (11 patients, 22.9%) was significantly less than that in the conventional group (12 patients, 57.1%) (*p* = 0.006) (Table 4).

**Table 4:**
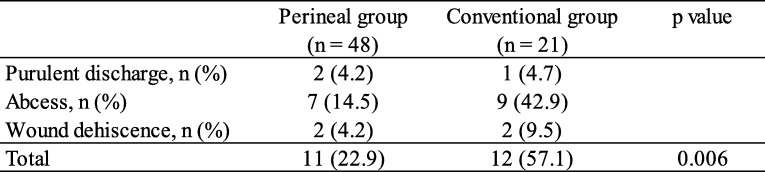
Perineal wound complications following surgery

### Oncological outcomes

Oncological outcomes are shown in Table 5. Complete TME was performed in all patients. Although 4 (19.0%) patients in the conventional group had a positive CRM at the anterior aspect, all patients in the perineal group had a negative CRM. Significant differences between the two groups were observed (*p* = 0.011).

**Table 5:**
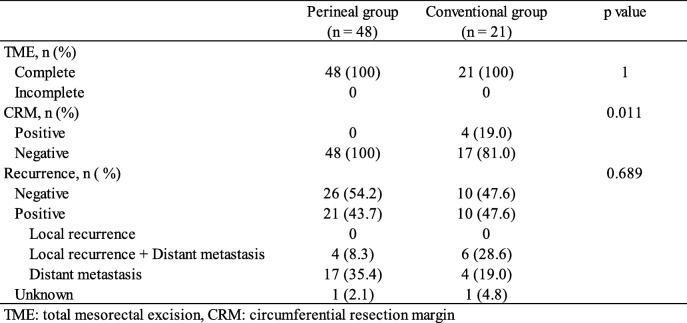
Oncological outcomes

Of the 69 patients, recurrence occurred in 31 (44.9%) patients in this series; both local recurrence and distant metastasis developed in 10 patients, and only distant metastasis developed in 21 patients. There were no significant differences between the two groups (*p* = 0.689); recurrence occurred in 21 (43.8%) patients in the perineal group and in 10 patients (47.6%) in the conventional group.

**Fig. 3:**
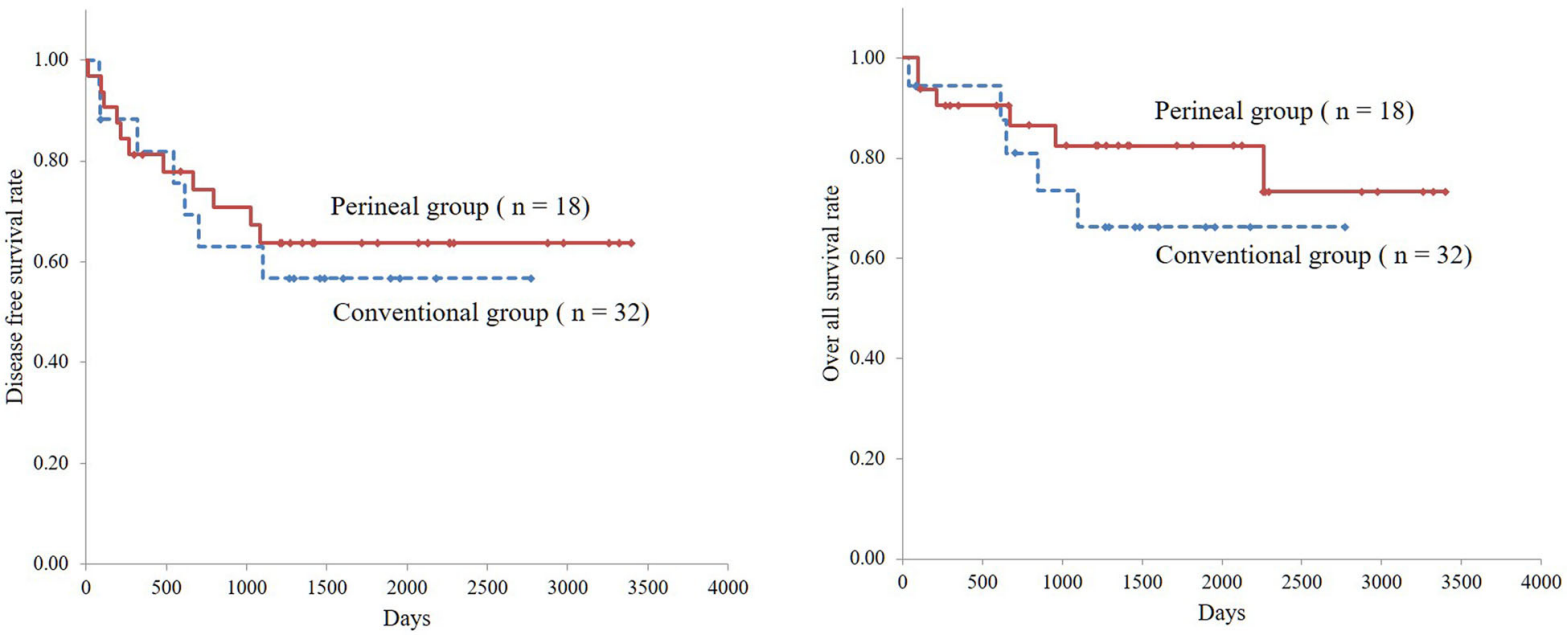
**a**: 5-year disease-free survival curves. **b**: 5-year overall survival curves

Excluding 15 patients with stage 4 cancer and 4 patients whose prognosis was not known, the 5-year disease-free survival rates in the perineal and conventional groups were 63.7 and 56.7%, respectively. Additionally, the 5-year overall survival rates in the two groups were 82.5 and 66.2%, respectively. There were no significant differences in 5-year disease-free survival and overall survival rates (*p* = 0.665 and 0.323, Fig. 3).

## Discussion

In rectal surgery, TME and negative circumferential resection margins are prerequisites for minimizing local recurrence after surgery [7–10]. Although laparoscopic surgery for rectal cancer has benefits compared with open surgery, laparoscopic surgery is still challenging in male sex, high body mass index, visceral obesity, a narrow pelvis, bulky tumor and an advanced T-stage [11, 12]. Actually, randomized controlled trials including the ALaCart [1] and ACOSOG Z6051 [2] trial failed to show the noninferiority of laparoscopic surgery compared with open surgery for oncologic outcomes. Recently, a new approach, the transanal mesorectal excision (TaTME) has been attracting attention as a promising technique for rectal cancer patients for whom laparoscopic TME may not be achieved completely [13–15].

Regarding preservation of sphincter function in rectal carcinoma surgery, historically, RA has been the preferred method for low-lying rectal cancer near the anus. Owing to ISR, sphincter-preserving resection has made great progress. According to Rullier et al., ISR made it possible to preserve the anus in patients with low-lying type-3 tumors near the anus [16]. However, there could be risk of local recurrence in super low anterior resection and ISR for low-lying advanced tumors near the anus. Using of titanium and braided sutures in anastomosis near the anus could provide a substrate for exfoliated malignant cells [17]. Also, Yamada et al. [18] reported the high local recurrence rate of 11.5% following ISR taken from the ISR questionnaire result in Japan. The pT factor, pN factor and the level of ISR were significant risk factors. In particular, the high local recurrence was significantly associated with patients with pT3 (invasion to the external anal sphincter muscle) and pT4. It is difficult to accurately diagnose the invasion depth of tumors for low-lying tumors near the anus preoperatively; therefore we have no choice but to diagnose the tumors during surgery. If conversion to RA from ISR is required because of unexpected tumor invasion of the external anal sphincter muscle during surgery, then RA with an initial perineal approach must be reasonable and may have benefits. However, there are no reports on the feasibility of this approach. In this study, we compared the surgical and oncological outcomes between the two RA approaches for primary anorectal cancer. Consequently, we found three advantages to the perineal approach, which should be the first RA technique for the selected primary anorectal carcinoma.

The first advantage was that the operation time in the perineal group was significantly shorter than that in the conventional group (313 vs. 388 min; *p* = 0.027). Laparoscopic surgery for RA remains challenging. It is very difficult to perform TME toward the pelvic floor laparoscopically, especially in males with a narrow pelvis and in patients with a bulky tumor located in the pelvis. In RA, a perineal retrograde anorectum dissection prior to the transabdominal maneuver might make RA easier and decrease the operation time. However, RA was performed laparoscopically for only 16 patients (12 patients in the perineal group and 4 patients in the conventional group) in this study.

The second advantage was that the occurrence of PWC was significantly lower in the perineal group than in the conventional group (22.9% vs. 57.1%; *p* = 0.006). In colorectal surgery, surgical site infection (SSI) was reported more frequently. Ata et al. [19] reported that SSI in colorectal surgery developed 3.8 times more often than SSI in noncolorectal general surgery. Additionally, the incidence of SSI in rectal surgery is higher than that in colon surgery. In particular, SSI following abdominoperineal resection (APR) is common [20]. In Japan, the SSI occurrence rate was reported to be 25–47% [21, 22]. The large amount of dead space in the pelvis following RA, closure under tension and the closure of a wound in an area that has a high bacterial count [23, 24] may be causes for the rate of SSI. In this series, PWC occurred in 33.3% of all patients who underwent RA for primary anorectal carcinoma.

Various risk factors such as smoking [25–27] hypoalbuminemia [28], ASA classification [25] obesity [29], weight loss [25], the number of comorbidities [30, 31] as a [27, 29], neoadjuvant radiation therapy/chemoradiation therapy [27, 32, 33] as a tumor-related factor, flap reconstruction [26, 27] and extralevator APR [34, 35] as an operation-related factor are reported risk factors for PWC after APR. Generally, SSI risk factors, including a prolonged operation time, extensive bleeding, intraoperative blood transfusion and other risk factors related to surgery, are well known. Shortening the operation time by starting with a perineal approach might have reduced the incidence of PWC in this study.

Traditionally, certain treatments, including omentoplasty, perineal mesh placement, and flap reconstruction, have been performed to reduce PWC incidence following RA [36]. More recently, negative pressure wound therapy (NPWT) for the perineal wound following RA was reported as a new wound management technique [37]. For primary closed perineal wounds following RA, NPWT was associated with a reduced incidence of perineal SSI compared with only a gauze dressing [38]. On the other hand, van der Valk MJM et al. [39] reported in a pilot study that incisional NPWT decreased the duration of wound healing but did not reduce the rate of wound complications. The significance of NPWT for PWC following RA is still unknown. On the other hand, the rate of postoperarive complications excluding PWC was similar between the two groups. Urinary complications did not increase by an initial perineal approach in RA.

The third advantage was that positive CRM was significantly lower in the perineal group than in the conventional group. CRM is an important aspect in minimizing local recurrence after rectal cancer surgery [33, 34]. In this series, although there were no patients with positive CRM in the perineal group, four male patients in the conventional group presented positive CRM. For these four patients, the tumor was located at the anterior aspect of the lower rectum. RA with an initial perineal approach has the advantage of surgical margin safety due to direct visual observation during rectum dissection, especially when the tumor is located at the anterior aspect of the rectum. The result in this study might be due to the advantage of this approach, but there was no difference in the rate of recurrence between the two groups.

There were some limitations in this study. First, the sample number of patients who underwent RA, especially those who underwent laparoscopic surgery, was very small; there were 12 patients in the perineal group and 4 patients in the conventional group. This was a retrospective and nonrandomized study. The results may have been affected by its retrospective design for selective primary anorectal carcinoma. Additionally, although possible PWCs were diagnosed by two surgeons who routinely conducted rounds after surgery to observe the wounds, there was a risk of underreporting the incidence of PWC following RA. Second, the decision of whether a patient should undergo the initial perineal approach or initial abdominal approach was not standardized. In this study, the surgical approach for RA was chosen according to surgeon’s preference. It was not known whether the decision was made based on tumor characteristics or other factors. The surgeon alone characterized the operation as either an initial perineal or initial abdominal approach.

## Conclusions

These results indicate that RA with an initial perineal approach for selective primary anorectal carcinoma might have advantages in minimizing PWCs and positive CRMs. To confirm the advantages of RA with an initial perineal approach, especially for laparoscopic surgery, a randomized study will be needed.

## Data Availability

The datasets used and/or analyzed during the current study are available from the corresponding author on reasonable request.

## Abbreviations

APR: Abdominoperineal resection
CA19–9: Carbohydrate antigen 19–9
CEA: Carcinoembryonic antigen
CRM: Circumferential resection margin
CT: Computed tomography
ISR: Intersphincteric resection
MRI: Magnetic resonance imaging
Pre-CRT: Preoperative chemoradiation therapy
PWC: Perineal wound complication
RA: Rectal amputation
TaTME: Transanal total mesorectal excision
TME: Total mesorectal excision
